# Tumor Treating Fields Perturb the Localization of Septins and Cause Aberrant Mitotic Exit

**DOI:** 10.1371/journal.pone.0125269

**Published:** 2015-05-26

**Authors:** Nidhi Gera, Aaron Yang, Talia S. Holtzman, Sze Xian Lee, Eric T. Wong, Kenneth D. Swanson

**Affiliations:** 1 Department of Biological Chemistry and Molecular Pharmacology, Harvard Medical School, Boston, Massachusetts, United States of America; 2 Department of Neurology, Division of Neuro-Oncology, Beth Israel Deaconess Medical Center, Boston, Massachusetts, United States of America; Institut de Génétique et Développement de Rennes, FRANCE

## Abstract

The anti-tumor effects of chemotherapy and radiation are thought to be mediated by triggering G_1_/S or G_2_/M cell cycle checkpoints, while spindle poisons, such as paclitaxel, block metaphase exit by initiating the spindle assembly checkpoint. In contrast, we have found that 150 kilohertz (kHz) alternating electric fields, also known as Tumor Treating Fields (TTFields), perturbed cells at the transition from metaphase to anaphase. Cells exposed to the TTFields during mitosis showed normal progression to this point, but exhibited uncontrolled membrane blebbing that coincided with metaphase exit. The ability of such alternating electric fields to affect cellular physiology is likely to be dependent on their interactions with proteins possessing high dipole moments. The mitotic Septin complex consisting of Septin 2, 6 and 7, possesses a high calculated dipole moment of 2711 Debyes (D) and plays a central role in positioning the cytokinetic cleavage furrow, and governing its contraction during ingression. We showed that during anaphase, TTFields inhibited Septin localization to the anaphase spindle midline and cytokinetic furrow, as well as its association with microtubules during cell attachment and spreading on fibronectin. After aberrant metaphase exit as a consequence of TTFields exposure, cells exhibited aberrant nuclear architecture and signs of cellular stress including an overall decrease in cellular proliferation, followed by apoptosis that was strongly influenced by the p53 mutational status. Thus, TTFields are able to diminish cell proliferation by specifically perturbing key proteins involved in cell division, leading to mitotic catastrophe and subsequent cell death.

## Introduction

Mitosis proceeds in highly choreographed stages that must be executed with exquisite fidelity in order to ensure that both daughter cells are genetically identical to the parent cell. Subsequent to the formation of the mitotic plate, the paired kinetochores of the newly synthesized sister chromatid are captured by the ends of microtubules of the opposing metaphase spindles aligning each chromatid towards their respective poles during anaphase followed by cytokinesis. Microtubule capture by the kinetochores produces tension across the center of chromosomal pairs. Prior to the production of this tension, non-captured kinetochores produce a signal that prevent the activation of Cdc20 which is required by Anaphase Promoting Complex C (APC/C) to target the ubiquitin-mediated destruction of proteins such as Cyclin B and Securin [1, 2]. Upon Cyclin B and Securin degradation, sister chromatids separate and the cell rapidly and irrevocably proceeds into anaphase [3–7] and cytokinesis [8]. Ingression of the cytokinetic cleavage furrow (CCF) is driven by non-muscle myosin II and is required to mechanically separate the forming daughter cells from each other [9–11]. During mitosis, myosin activation within the CCF is linked to metaphase exit by its dependence on APC/C^Cdc20^ activity and its formation is directed by proteins located within the anaphase spindle midline, which contains proteins critical for its RhoA-dependent activation [12–14]. Therefore, the two hallmarks of anaphase, chromosome separation and activation of the contractile elements within the CCF are driven in parallel downstream of final kinetochore capture and APC/C^Cdc20^ activation. This in turn is dependent on proper microtubule function within the metaphase and anaphase spindles. Unlike errors or damage that initiate the G_1_/S, G_2_/M or spindle assembly check point (SAC), with the possible exception of errors involving failures in chromatid separation [15, 16], catastrophic errors that occur after the cell has committed to anaphase are unlikely to be correctable [8]. Therefore, errors committed at this point in the mitosis lead to mitotic catastrophe and aberrant mitotic exit and/or cell death.

The activation of non-muscle myosin II within the CCF is governed by the small G protein, RhoA, whose localization and activation is determined by proteins bound to the midline of the rapidly formed anaphase spindle [17]. To accomplish this, the anaphase spindle midline contains the centralspindlin complex (composed of KIF23 and MgcRacGAP) which is phosphorylated by resident PLK1 to create binding sites for the RhoGEF ECT2. In this way, ECT2 is recruited it to the midline [18] from where it is subsequently delivered to the CCF [19]. ECT2 binds the adaptor protein Anillin which in turn binds to the Septin 2, 6 and 7 heterotrimer [20] to recruit them to late M-phase structures. ECT2 is not only instrumental in directing the localization and regulation the CCF function but, along with Anillin, is required for the stability of the anaphase spindle midline which becomes disordered upon the depletion of either of these proteins [21]. During mitosis, the Septin protein complex is also recruited to both the anaphase spindle midline and cleavage furrow [22] where it likely cooperates with Anillin to participate in stabilizing both the microtubule structure of the anaphase spindle and demarcates the boundaries of the cleavage furrow contractility [20, 21, 23, 24]. The Anillin/Septin complex is also recruited to the CCF where it plays an essential structural role by cross-linking actin, non-muscle myosin II [25] and RhoA [17], facilitating their interaction and promoting actin-based myosin contraction that drives cleavage furrow ingression. In addition, Anillin and Septin restrain this myosin activity to within the furrow and thus confines contraction to the equatorial plane that overlays the fission plate [26]. Depletion or mutation of ECT2 [27], Anillin [17] or Septin 2, 6, or 7 [28, 29] has been shown to result in defective cytokinesis leading to violent plasma membrane blebbing and spindle rocking. These findings strongly suggest that perturbation of structural/regulatory elements within this pathway results in a common endpoint of mitotic catastrophe during anaphase.

Studies of responses to drugs that force mitotic catastrophe such as microtubule or Kinesin V motor poisons have illustrated that interference with processes involved in the transition from metaphase to anaphase results in a variety of outcomes ranging from normal cell division to mitotic slippage which is mitotic exit in the absence of cytokinesis [30–33]. Such defects in CCF function can result in uneven segregation of chromosomes resulting in aneuploidy [34]. Such events have been shown to induce a p53-dependent checkpoint in the subsequent G_0_ or G_1_ phases of the cell cycle [35, 36] due to the presence of damaged spindle elements, supernumerary centrioles and tetraploidy [37]. Therefore, perturbation of key mitotic proteins in anaphase results in significant cellular damage and derangement.

It has recently been demonstrated that alternating electric fields referred to here as Tumor Treating Fields (TTFields), are able to disrupt cells during mitosis within a narrow range of frequencies with peak activity between 100 and 200 kHz; such fields have little measureable effect on cell biology at frequencies of 500 kHz or above, [38]. Exposure of cells to these non-ionizing, alternating electric fields can have different consequences depending on the frequency of electric fields. For instance, at 1 kHz or lower, alternating electric fields cause depolarization of membrane potentials in excitable cells, such as neurons, cardiac myocytes, and muscle cells, via the opening of the voltage-gated ion channels [39–41] Defibrillators and electro-shock therapy both rely on this ability of high intensity electric fields to induce membrane depolarization. It is thought that TTFields exert dielectrophoretic forces on cellular components [42] and alter their normal functions. Specifically, under cell culture conditions, these forces affected cells during division in a way that resulted in the induction of violent plasma membrane contractions during mitosis and cell death [38, 43]. When applied from outside of the body in animal models, the alternating electric fields resulted in tumor regression in tumor models [43–45]. A therapeutic device designed to deliver such electric fields to humans, the NovoTTF-100A, has been approved by the FDA for the treatment of recurrent glioblastoma based on a successful phase III clinical trial [46, 47] However, the mechanism by which these electric fields perturb cells during mitosis and their long term effects on cells that have survived this insult may underlie these therapeutic effects is not completely understood.

We have found that the initiation of membrane blebbing caused by TTFields occurs within mitosis at times coincident with the expected onset of anaphase. These membrane contractions were likely due to aberrant localization and/or function of the contractile elements, resulting in ectopic furrow formation. As in the case with spindle poisons, which trigger the SAC, cells affected by TTFields exhibit different fates including death in anaphase or aberrant exit from mitosis similar to mitotic slippage. Septin localization to the midline of the anaphase spindle and cleavage furrow, as well as its re-association with microtubules upon cell spreading, were perturbed by TTFields which strongly suggests that their effect on cells during mitosis is due to their ability to perturb the localization and/or function of the protein complexes known to regulate CCF function. Given the critical role played by Septins in the formation and regulation of the CCF during cell division, we propose a mechanism of action where TTFields perturb mitosis by interfering with normal Septin localization and function leading to CFF dysfunction and aberrant mitotic exit. Finally, cells affected by TTFields exposure during mitosis subsequently exhibited p53-dependent decrease in proliferation and an increase in apoptosis. These data demonstrate that TTFields perturb mitotic cells within anaphase by a mechanism unrelated to previously known therapeutic interventions and result in cellular derangement leading to decreased proliferation or death and provide evidence that patient tumor genetics may influence the clinical response to TTFields treatment.

## Materials and Methods

### Antibodies and reagents

Anti-(Ser10) phospho-histone H3, Anti-cyclin B, Anti-α-tubulin and anti-p53 antibodies were purchased from Santa Cruz (Santa Cruz, CA). Anti-Securin and anti-PLK1 were purchased from Abcam (Cambridge, MA). DRAQ5 and p38 was purchased from Cell Signalling Technologies (Danvers, MA). Anti-Septin 7 was purchased from Proteintech Group. Annexin V, and 7-AAD were purchased from BD Biosciences (San Jose, CA). Caspase-3 and -7 Assay Kit, DAPI, rhodamine-labeled phalloidin, Alexa 488 and Alexa 564-conjugated secondary antibodies was purchased from Life Technologies (Foster City, CA). R03306, Aphidicolin, Paclitaxel and Fibronectin were purchased from Sigma-Aldrich, (St. Louis, MO). Myosin-IIA-GFP was purchased from Addgene (Cambridge, MA).

### Cell culture

Cultures of HeLa, MDA-MB-231, HCT116 and MCF-7 were originally purchased from ATCC (Manassas, VA), the p53^+/+^ and p53^-/-^ HCT116 cell lines were a gift from Dr. Bert Vogelstein and were cultured in RPMI 1640 medium containing 10% FCS and incubated at 37°C and 5% CO_2_. All other cell lines were cultured in DMEM supplemented with 10% FCS. Cells were synchronized by double aphidicolin block. Briefly, cells were treated with 9 μM aphidicolin for 16 hrs, trypsinized and re-plated and then seeded onto either 22mm Thermanox tissue culture plastic (Nunc) or 22 mm number 1 glass cover slips, incubated for an additional 6 hours and treated with either 3 μM aphidicolin or 9 μM RO3306 CDK1 inhibitor for an additional 16 hrs prior to removal [48]. For TTFields treatment, 10,000 to 20,000 cells were plated allowed to adhere to tissue culture plastic or glass and exposed to fields of 1 v/cm2 at either 150 kHz or 500 kHz at a current of 150 mA generated by Inovitro TTFields generators (NovoCure Inc., Haifa, Israel) as described previously [38].

### FACS analysis

FACS was performed with indicated antibodies incubated in FACS buffer (PBS containing 0.5% BSA and 0.1% sodium azide) for 1 hr and fluorophore labeled secondary of 30 min. At least 10,000 cells per condition were analyzed using FACSCalibur (BD Biosciences, San Jose, CA). For intracellular staining, cells were fixed using 4% paraformaldehyde in PBS and then permeabilized in 70% methanol at -20°C overnight prior to rehydration in PBS and staining with the indicated antibody.

### Microscopy

#### Immunofluorescence and florescent protein localization

Cells were plated onto glass cover slips and treated as indicated. For phalloidin staining, aphidicolin-synchronized cells were plated onto poly-L-lysine coated cover slips and fixed in cytofix buffer (20% sucrose, 129 mM KCl, 20 mM Pipes pH 6.8, 2 mM EDTA and 4% PFA) for five minutes immediately following TTFields exposure during mitosis. For immunoflourescence, cells were fixed and permeabilized with 100% ice cold methanol and incubated at -20°C, rehydrated in PBS and blocked overnight in PBS with 1% normal goat serum and stained with the indicated antibodies in blocking buffer containing 3% BSA. Bound antibodies were visualized using Alexa 488 or Alexa568-conjugated anti-rabbit IgG or anti-mouse IgG (Invitrogen). Wide field images were obtained using a Zeiss Axiovert 200M microscope with an oil immersion 63X Plan-Apochromat objective. Images were analyzed using Axiovision software. Confocal images were obtained with a Zeiss AX10 microscope equipped with a 63X Plan Apochromat objective lens. Quantification of Septin and F-actin localization was performed using Metamorph software. The average intensity of Septin staining at the midline was normalized with whole cell average intensity. Myosin-IIA-GFP protein was transiently expressed in HeLa cells. Cells were synchronized overnight with aphidicolin and treated as described. Cells were fixed by 4% PFA, and stained with/DAPI. Images were acquired using Nikon Ti w/ A1R confocal microscope equipped with a 60X Plan apochromat objective lens. Images were produced by using Nikon Elements software.

#### Live cell imaging

Cells were plated onto glass bottom dishes and synchronized using 3 μm aphidicolin for 16 hours. The aphidicolin was removed and cells were allowed to progress to M phase. Cells were incubated in 6 μm DRAQ5 for 30 minutes and then washed twice in phenol free DMEM containing 10 mM HEPES pH 7.4. Cells were imaged at 200X using a Nikon Ti inverted microscope with Perfect Focus System equipped with a Hamamatsu ORCA-R2 CCD camera and images were processed using Metamorph 7 software.

#### Post-TTFields cell counts

Cells were synchronized by double aphidicolin block and seeded on 35 mm plates possessing gridded cover slips on their bottoms. After the drug was washed out, the cells were treated or left untreated at 37°C for 16 hr during mitosis, and then removed from the TTFields. Cells were counted within individual grids at 4 and 20 hours after removal from the TTFields and a ratio of the cells counted at 20 vs. 4 hr for each of the grids was calculated. For subjective scoring of Septin 7 microtubule localization, cells stained for Septin 7 and α-tubulin were assigned a subjective value based on association of Septin 7 with microtubules (scored as 3) vs diffuse within the cytoplasm (scored as 1) with intermediate cells being scored as 2 by two independent and blinded scorers.

## Results

### Cells treated with TTFields exhibit differences in mitotic progression

**Fig 1:**
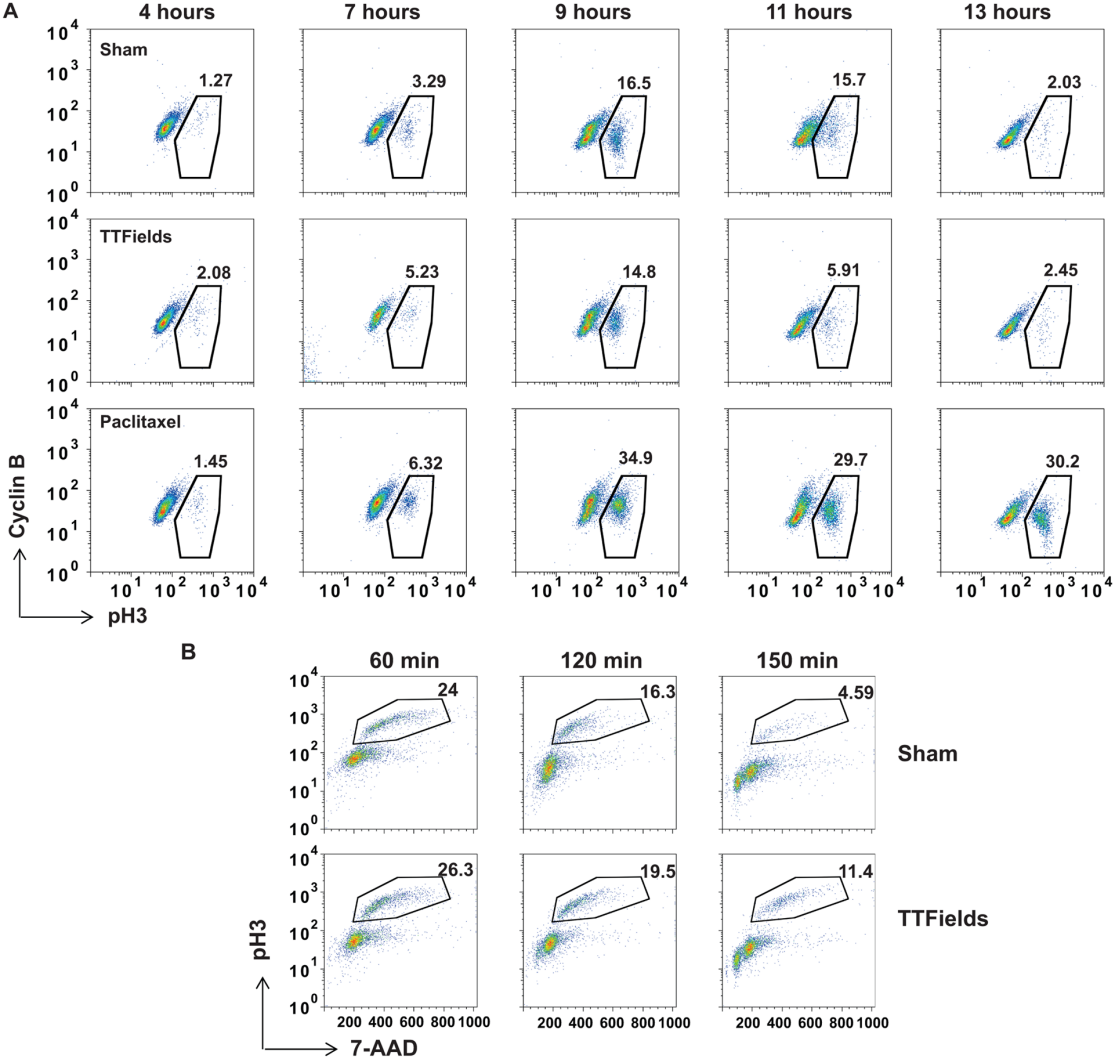
Cells exposed to TTFields exhibit an inability to progress through anaphase successfully resulting in cellular derangement. MDA-MB-231 cells were synchronized using double aphidicolin block and then allowed to re-enter the cell cycle in the absence of TTFields (top panels), exposed to TTFields (middle panels) or treated with 3 μM paclitaxel (bottom panels). Cells were harvested and analyzed by FACS for transit through mitosis by staining with antibodies against Cyclin B antibodies and pH3 at 4, 7, 9, 11 and 13 hours after aphidicolin removal. Cells treated with TTFields did not exhibit a marked accumulation of mitotic cells showing a slight increased accumulation of cells at the 9 and 11 hour time points compared to control cultures suggesting a reduction in the rate of mitotic exit (**A**). Results are representative of 4 separate experiments. MDA-MB-231 cells were synchronized using aphidicolin followed by RO3306 treatment and were then allowed to progress through mitosis n the presence or absence of TTFields following drug removal. Cells were then collected at 60, 120 and 150 min and stained with antibodies against pH3 and 7-AAD for DNA content for analysis by FACS (**B**).

Previous studies showed that cells exposed to TTFields exhibited violent plasma membrane contractions during mitosis, increased time in mitosis and decreased cellular accumulation in exposed cultures [38]. In order to better characterize the effects of TTFields on cell division, we synchronized MDA-MB-231 cells in S phase using aphidicolin and followed their progression through mitosis. Cells were harvested at 4, 7, 9, 11 and 13 hours following removal of the drug, were fixed and analyzed for Cyclin B and phospho-histone H3 (pH3) levels by FACS. Parallel cultures were either left untreated or treated with paclitaxel to block cells in metaphase. TTFields-treated cells largely transited through mitosis at rates similar to control cells whereas paclitaxel-treated cells exhibited a significant early and persistent accumulation of double positive cells (Fig 1A). Similar to other studies [38], we noted a small increase in mitotic cells at later time points in the TTFields-treated cultures relative to control cultures demonstrating an increase in time in mitosis. Therefore, in order to better examine the dynamics of mitosis within M-phase, we synchronized MDA-MB-231 cells using Cyclin B/CDK1 inhibitor RO3306 to block the cells from entering mitosis [48]. After drug removal, cells were treated with TTFields and individual cultures were removed from the treatment fields at 60, 120 and 150 minutes and stained for pH3 content and DNA using 7-Aminoactinomycin D (7-AAD) to follow mitotic progression. Cells exposed to TTFields persisted longer in mitosis and possessed a higher DNA content for a longer period relative to non-TTFields-treated control cells (Fig 1B). These data revealed that the TTFields-induced perturbation occurs during of the later stages of mitotic progression.

#### TTFields-treated cells exhibit mitotic membrane contractions coincident with metaphase exit

**Fig 2:**
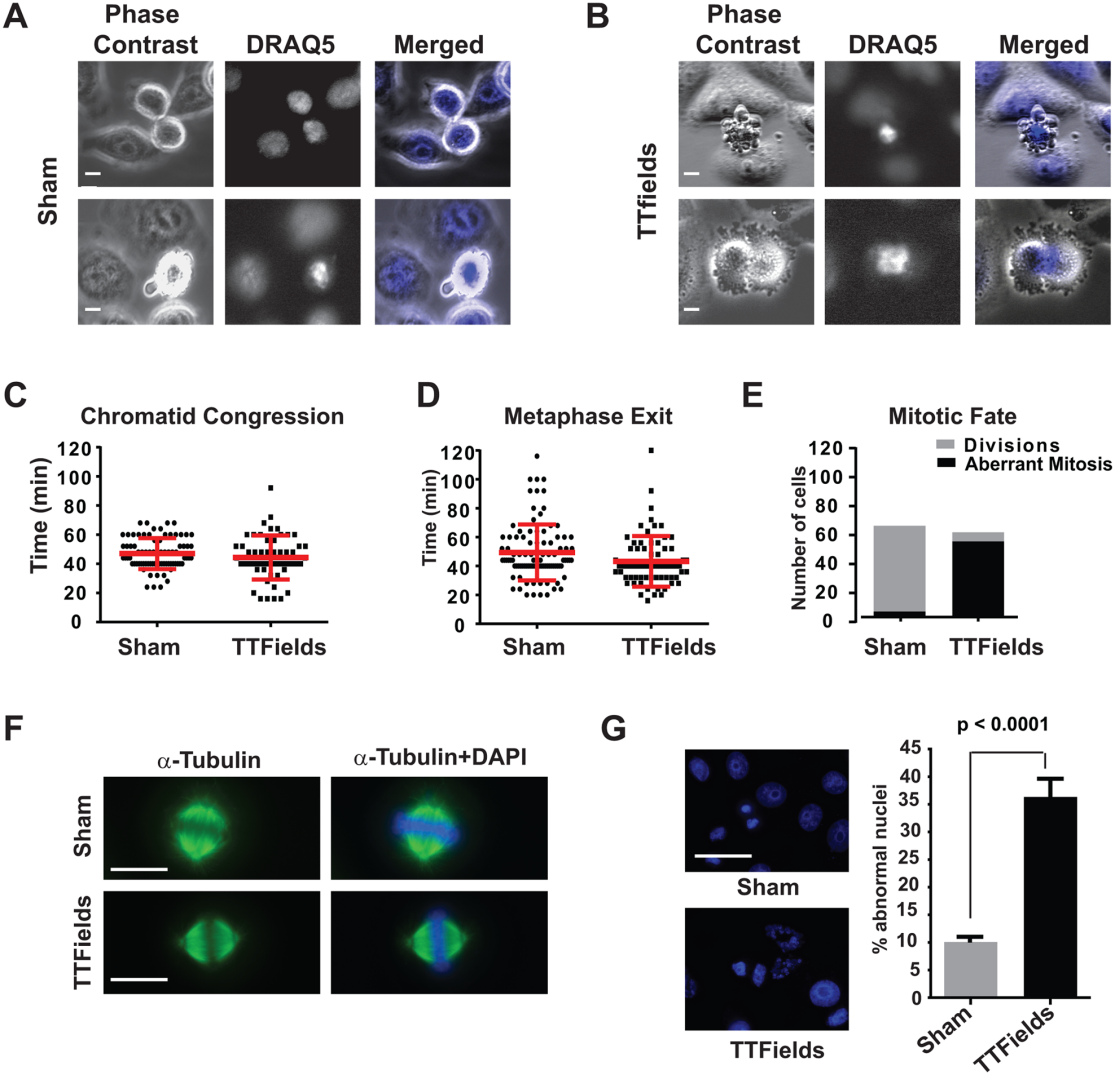
Cells exposed to TTFields during M-phase exhibit chromosomal disordering during the metaphase to anaphase progression. HeLa cells were partially synchronized by treating with aphidicolin, then stained with DRAQ5 to visualize their chromosomes and subjected to fluorescence and phase contrast time lapse microscopy. Cells were imaged as they transited through mitosis either with or without TTFields exposure by both phase contrast and fluorescence during exposure to TTFields and time lapse series were captured. Single frames extracted from the time-lapse series of either Sham-treated (**A**) or TTFields-treated (**B**) cells visualized by phase contrast (left panels) or DRAQ5 (middle panels) at intervals of 240 seconds showed cells undergoing violent mitotic contractions that appeared coincident with the separation of daughter chromosomes at the onset of anaphase. Measurement of the time intervals between chromatid condensation and formation of the metaphase plate (**C**) and from the formation of the metaphase plate to either evidence of anaphase or TTFields-induced membrane contractions (**D**) were similar, 49.39 ± 1.988 min vs. 43.26 ± 2.088 min for plate formation and 47.04 ± 1.196 min vs. 44.36 ± 2.037 min for metaphase exit in Sham-treated vs TTFields-treated cultures, respectively. However, the outcome of mitosis was markedly different in Sham-treated (n = 130) vs. TTFields-treated (n = 85) cultures (**E**). Mitotic spindles and metaphase plates formed normally in TTFields treated cells (**F**), however, cells exiting mitosis in TTFields-treated cultures exhibited increased in abnormal nuclei with many cell possessing multiple micronuclear structures (**G**). Scale bar = 20 μm.

The above data suggested that TTFields perturbed cellular function late during mitosis. Therefore, in order to better define the timing of the previously observed TTFields-induced perturbation, we fluorescently labeled the chromosomes of aphidicolin-synchronized HeLa cells with DRAQ5. Cells were then subjected to time-lapse microscopy following removal of the drug and imaged under both phase and fluorescence time-lapse microscopy either in the absence (Fig 2A) or presence of TTFields (Fig 2B). The chromosomal behavior within these cells showed that in both control and TTFields-treated cultures, cells formed metaphase plates at similar rates (Fig 2C). However, TTFields-treated cells exhibited membrane disruption and blebbing at times following mitotic plate formation that corresponded closely to the time of metaphase exit in control cultures (Fig 2D) with the chromosomes becoming disordered (Fig 2B and 2E, and S1 Movie vs. S3 Movie and S4 Movie and S1 Fig).

**Fig 3:**
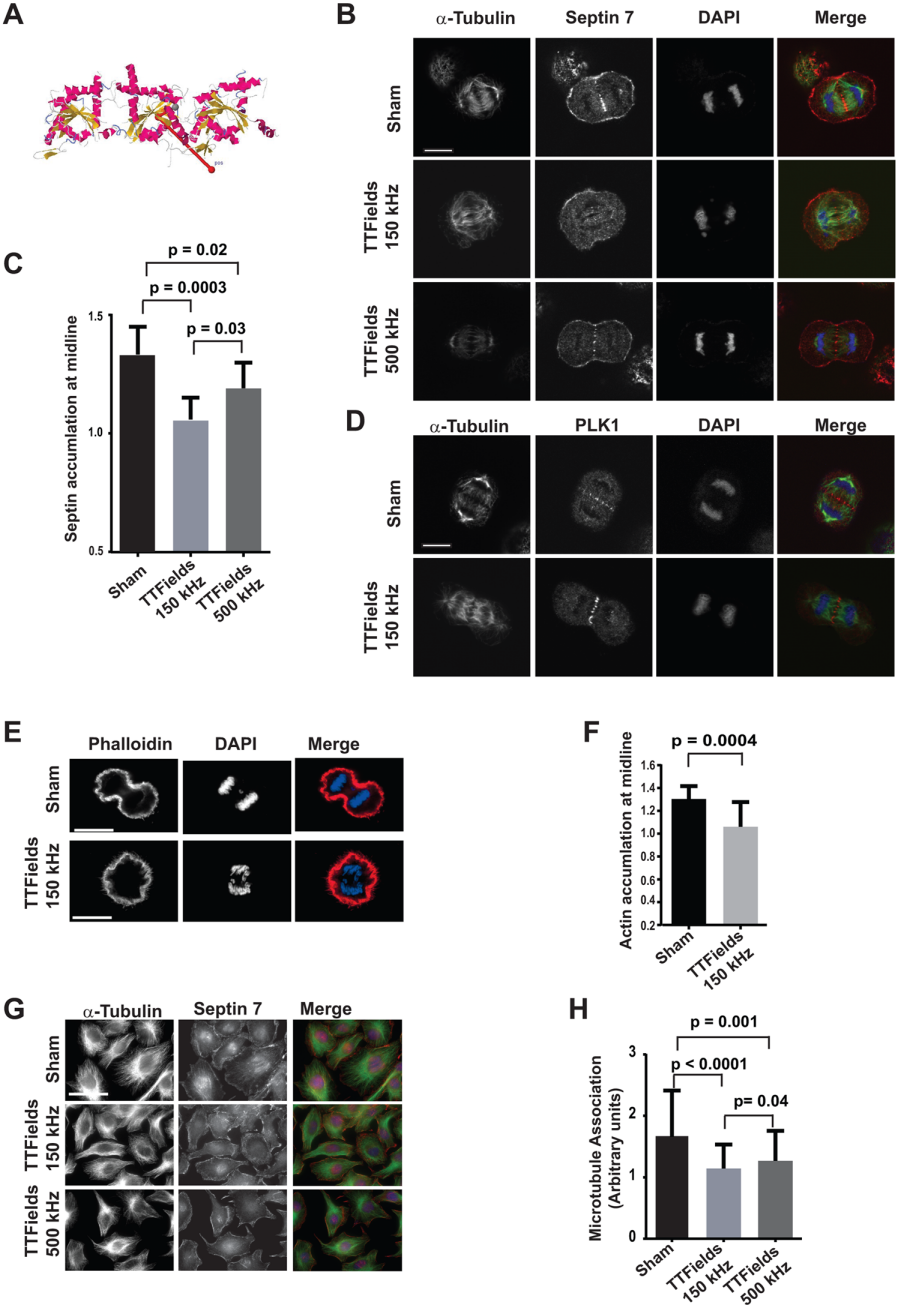
Septin 7 localization is perturbed by TTFields. Crystal structure of Septin complex composed of Septins 2, 6, and 7 showing the direction of the dipole vector (2711Debyes) relative to the longitudinal axis of the heterotrimer (**A**). Synchronized cells were cultured in the absence of TTFields, TTFields delivered at 150 kHz (n = 30) or 500 kHz (n = 12), fixed and stained with antibodies against α-tubulin and Septin 7 and counter stained with DAPI. Confocal microscopy of cells captured in mid anaphase under these conditions revealed that Septin 7 localization was reduced and the midlines of anaphase spindles were perturbed by TTFields of 150 kHz but to a lesser extent at TTFields at 500 kHz compared to control cells (n = 27) (**B**). The average intensity of Septin 7 staining was separately determined within the region of the midline and for the entire cell. The ratio of the average intensity of Septin 7 staining at midline that of the total staining within the cell in Sham (n = 10) and presence of TTFields at 150kHz (n = 9) or 500kHz (n = 8) showed a significant decrease in Septin accumulation at the midlines in cells treated with 150 kHz TTFields (**C**). In contrast to Septin 7, PLK1 localization to the anaphase spindle midline appeared unperturbed by TTFields at 150 kHz (n = 11) (**D**). Scale bar = 10 μm. Septin organizes filamentous actin within the CCF during anaphase, in 150kHz TTFields treated cells F-actin accumulation were significantly decrease at the midline (E and F), n = 16 sham and n = 17 TTFields. To test whether TTFields interfered with the ability of Septins to re-localize to interphase microtubules during attachment and spreading on fibronectin, cells were allowed to spread for 9 hours under Sham conditions, or treated with TTFields at either 150 kHz, or 500 kHz (**G**). The Septin localization to the cytoskeleton was scored based on the striation of Septin 7 at the base of the cell with 1 equating to no diffuse staining with no striations, 2 equating to moderate localization and striations, and 3 equating to strong striations. While cells were allowed to re-attach and spread, cells in the absence of TTFields exposure exhibited strong Septin 7 association with microtubules (n = 70) while cells that were exposed to TTFields at 150 kHz (n = 152) exhibited significantly less Septin 7 localization to microtubules. Cell spreading while exposed to TTFields of 500 kHz (n = 104) exhibited an intermediate amount of microtubule association. Data was accumulated from three separate experiments (**H**). Scale bar = 50 μm.

The behavior of cells attempting to divide while exposed to TTFields was not uniform. While most cells began to exhibit membrane contractions at a time consistent with the exit from metaphase (see Fig 2B upper panels), others showed defective furrow formation resulting furrow regression and rapid coalescence into a single binucleated cell (see Fig 2B lower panels) and a small number of cells successfully divided into daughter cells. We also observed that Sham-treated cells also exhibited membrane blebbing, albeit at significantly lower rates (see Fig 2A lower panels and Fig 2E and S2 Movie). These blebs were also resolved at a faster rate than for those seen in cells affected by TTFields, suggesting differences in how they were generated (S3 Fig and S5–S8 Movies). Staining mitotic cells for α-tubulin and DAPI revealed that the metaphase spindles of cells undergoing mitosis while exposed to TTFields appeared grossly normal (Fig 2F) while examination of RO3306-synchronized cultures shortly after the completion of mitosis revealed the presence of large numbers of cells with aberrant nuclear architecture, such as increased binucleation and the presence of cells with multiple micronuclei (Fig 2G). We also noted that M-phase cells within the field that were able to progress into anaphase exhibited disordered anaphase spindles (see Fig 3B and 3C). These timing of these observations strongly suggested that TTFields interfered with the proper formation and/or regulation of the CCF.

#### TTFields perturb Septin localization

TTFields are able to affect cellular physiology through their effects on proteins that both possess high dipole moments and are also critical to specific cellular functions, in this case mitosis [38]. Since we observed clear evidence that the TTFields-induced membrane blebbing that started coincident with the expected time of anaphase, we therefore reasoned that TTFields exerted their effects on mitosis by imposing rotational or torsional stresses on a protein or proteins involved in CCF formation and/or regulation. The septin protein complex consisting of Septins 2, 6 and 7 is recruited to both the anaphase spindle midline and cleavage furrow where it cooperates with Anillin to both stabilize the microtubule structure of the anaphase spindle and demarcate the boundaries of the cleavage furrow contractility [20, 21, 23, 24]. Significantly, the depletion of either Anillin [17] or Septin 6, or 7 [28, 29] through knockdown resulted in membrane blebbing similar to that seen in TTFields-treated cells [28]. We therefore considered the Septin complex as a potential target of the TTFields in mediating this mitotic disruption.

Using the online protein dipole moments calculation utility at the Weizmann Institute (http://dipole.weizmann.ac.il/) [49] and the crystal structure for the septin heterotrimer (PDB 2QAG) [50], we found that this protein complex possessed a high dipole moment of 2711 Debyes (D) with a directional vector that is orthogonal to the longitudinal axis of the complex (Fig 3A). Significantly, this dipole moment is approximately five standard deviations higher than the median value of 542.66 D derived from the analysis of 14,960 protein structures [49]. Further, these heterotrimers further oligomerize to form larger hexamers [50]. Interestingly, the orientation of the dipole vectors of the constituent heterotrimeric subunits is predicted to be in roughly the same plane and direction in each bound subunit in the hexamer. Therefore, it is likely that the total dipole of this hexamer is roughly the sum of the heterotrimers thereby increasing the ability of TTFields to act on it.

The septin complex forms filaments associated with the microtubule cytoskeleton during interphase [51, 52] and diffuses within the cytoplasm upon cell rounding and microtubule de-polymerization (not shown). As mitosis progresses, the Septin 2, 6 and 7 heterotrimer localizes to the plasma membrane, anaphase spindle midline and the CCF. Synchronized cells were exposed to TTFields delivered at 150 kHz during mitosis and fixed and stained for α-tubulin and Septin 7. Their DNA was stained with DAPI to characterize stages of mitosis. We observed little reproducible difference in Septin 7 localization between control and TTFields-treated cells during early metaphase (not shown). However, in TTFields-treated cells that were imaged subsequent to their entry into anaphase, there was a marked diminution in Septin localization to the midline of the anaphase spindle and CCF (Fig 3B). Quantification of Septin localization to midline structures in Sham- and TTFields-treated cells revealed that cells dividing in the presence of both 150kHz and 500kHz TTFields exhibited a significant decrease of Septin 7 staining (Fig 3C). This demonstrates that TTFields were able to alter septin behavior. As expected we also observed a significant difference in the ability of septin to localize in the midline of cells treated with 150kHz TTFields when compared to those treated with 500kHZ TTFields where the latter exhibited an intermediate amount of septin localization compared to the sham- and 150 kHz treated cells which is consistent with the increased viability reported for cells treated at this frequency [35].

In addition to the perturbation of septin localization in TTFields-treated cells, the anaphase spindle midlines present in TTFields-treated populations were profoundly disordered (Fig 3B and 3C). However, since we detected similar levels of PLK1 within the anaphase spindles of TTFields-treated cells, this demonstrated cells undergoing mitosis within TTFields were able to form anaphase spindle midline structures even though Septins failed to localize to them (Fig 3D). Cells treated with alternating electric fields at 500 kHz exhibited an intermediate level of effect on Septin localization suggesting that these partial levels of Septin activity may be sufficient to support mitotic function in cultured cells (Fig 3B and 3C).

Anillin is delivered to the CCF where it normally binds to both Myosin II and Septin [20, 25] examination of cells expressing GFP-labeled myosin II showed that it is recruited to the CCF in both sham and TTFields-treated cells. However, it appears to be more disordered in the TTFields treated cells which exhibited fibrous and punctuate structures outside of the furrow (S2 Fig). Septins are responsible for the organization of F-actin within the CCF and bundles F-actin [53] as found in the submembranous cellular cortex which are necessary to drive successful cellular division. Therefore the failure to properly localize septin during mitosis would be predicted to result in loss of F-actin accumlation at midline and result in mitotic failure. Indeed, F-actin accumulation was significantly decrease in TTFields-treated cells as compared to sham-treated cells which further supports a role for Septin perturbation in TTFields-induced mitotic failure (Fig 3E and 3F).

Despite the septin complex’s high dipole moment and similar effects of Septin knockdown and TTFields exposure on mitosis, it remained possible that the TTFields effect on Septin was indirect. We therefore examined other processes where Septin localization might be perturbed by TTFields. During interphase, the Septin heterotrimer is found in filaments that are recruited to microtubules by microtubule associated protein-4 (MAP-4) [54] rather than Anillin. Septin becomes diffused within the cytoplasm upon suspension by trypsinization as microtubules depolymerize and becomes re-associated with them along with their re-formation upon cell spreading [51]. Consistent with our observations in cells undergoing mitosis, TTFields-treated cells re-plated in the presence of the TTFields exhibited a diffuse staining pattern with reduced cytoskeletal association compared to cells under control conditions. Consistent with our results in mitotic cells, cells forced to spread in the presence of 500 kHz alternating electric fields exhibited an intermediate level of Septin localization to microtubules (Fig 3E and 3F). Since microtubule association is mediated by MAP-4 and its recruitment to mitotic structure is driven by Anillin, these data provide strong support to the likelihood that TTFields affect mitosis by directly perturbing the mitotic function of the septin complex leading to the disruption of the anaphase spindle midline structure and CCF function.

### TTFields-treatment during mitosis results in cellular stress and reduced proliferation

**Fig 4:**
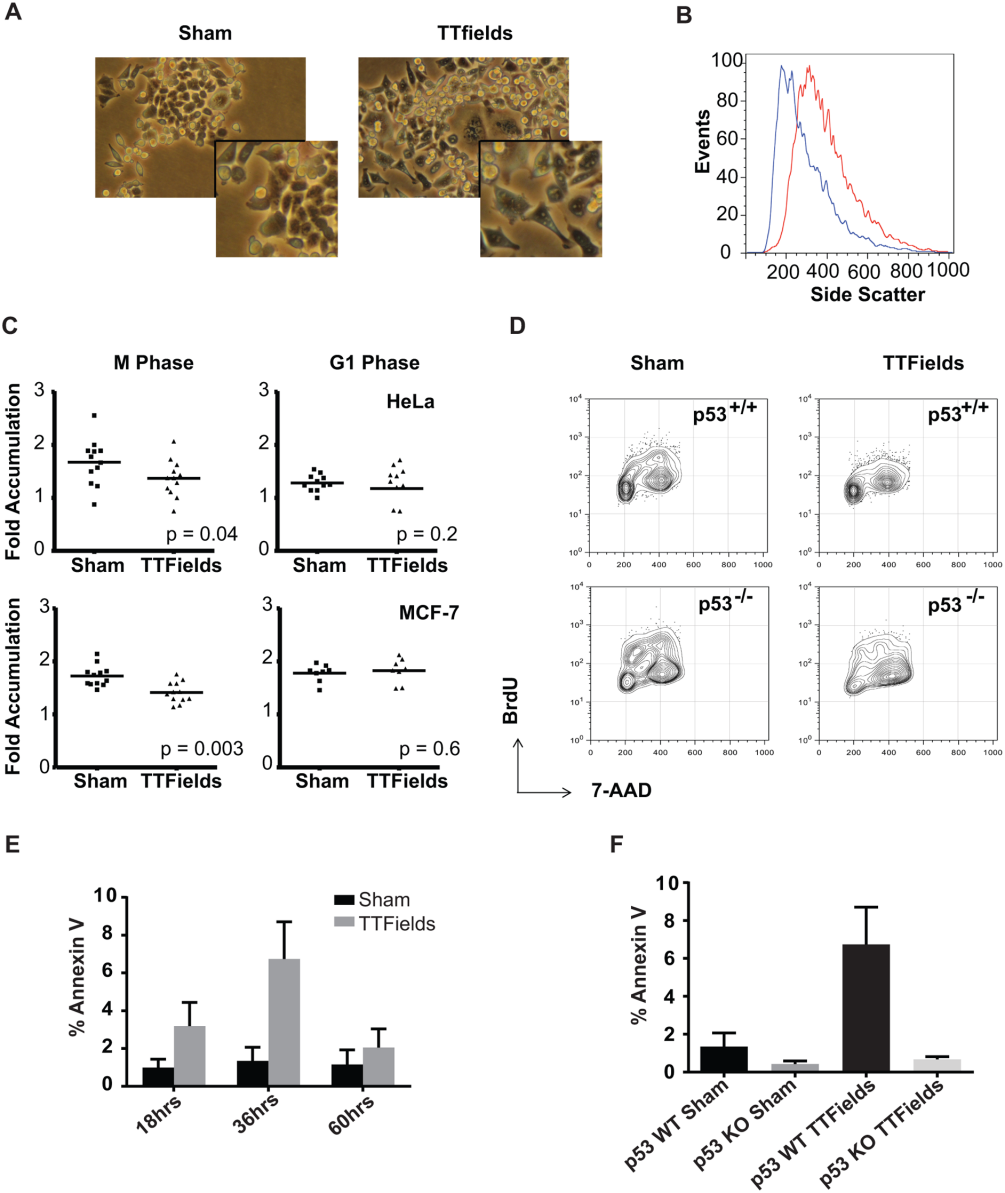
Cells exposed to TTFields often result in mitotic disruption and subsequent cell death. Upon their removal from the TTFields (right panel), cells exhibited significantly signs of cell stress including altered morphology compared to sham-treated controls (left panel) including increased size and vacularity (**A**). Insets show enlargement of cells in the field for detail. This increase in vacularity was also evident by increased side scatter by FACS (**B**). HeLa (upper panels) or MCF-7 cells (lower panels) were synchronized using aphidicolin, plated on gridded glass bottom dishes and either Sham-treated or exposed to TTFields during either mitosis or the G_1_ phase. After removal from the TTFields, cells were counted in individual grids at 4 and 24 hours after the termination of treatment and the resulting ratios were measured as a metric of proliferation. Most cells present at 4 hours remained at 24 hours. The proliferation of cells was significantly lower following exposure to TTFields during the M phase compared to sham-treated cultures. Both the TTFields-treated and Sham-treated cells exhibited similar proliferation when treated in G_1_ (**C**). HCT116 p53^+/+^ (upper panels) or HCT116 p53^-/-^ cells (lower panels) were incubated for 24 hours either without (left panels) or with TTFields-exposure (right panels) and then incubated for an additional 24 hours. Cells were allowed to incorporate BrdU into their DNA as a measure of cells in S phase (**D**). To test if cells exposed to TTFields exhibited a higher incidence of apoptosis. HCT-116 p53^+/+^ cells were treated with TTFields for 24 hours and then further incubated at 37°C and then stained with FITC-labeled Annexin V at 18, 36, and 60 hours after the midpoint of their treatment. Annexin V binding to cells was visualized by fluorescence microscopy and scored for the presence of Annexin V positive cells. Cells were observed to undergo apoptosis after 18 hours of removal from TTFields with a peak at 36 hours (**E**). In order to test the effect of p53 depletion on TTFields-induced apoptosis, the responses of HCT-116 p53^+/+^ were compared with HCT116 p53^-/-^ cells at 36 hours following TTFields treatment. p53^+/+^ with exposure to TTFields exhibited higher levels of apoptotic cells than their p53^-/-^ counterparts (**F**).

Aberrations in anaphase leading to tetraploidy or uneven chromosomal inheritance would be expected to result in a loss of viability in the post-mitotic cells. Subsequent to exposure to TTFields, cells exhibited morphologic changes that indicated cellular stress, including differences in cell shape and size and increased vacularity (Fig 4A and 4B). Therefore, in order to test whether TTFields exposure during mitosis resulted in post-mitotic consequences in these populations, synchronized HeLa and MCF-7 cells were plated on gridded dishes and exposed to TTFields during either M-phase or G_1_. Cultures were treated for 8 hours with TTFields within M phase. Images of cells on individual grids were then taken 4 hours after removal from the TTFields and then 24 hours later and the ratio of cell numbers between these two time points was calculated (Fig 4C left panels). This analysis showed a significant decrease in subsequent cellular accumulation when cultures were exposed to TTFields during M phase. However, such an affect was not seen when cultures were exposed to TTFields for the same period of time during G_1_ (Fig 4C right panels). MCF-7 cells were selected because they become larger and more spread upon the induction of tetraploidy which results from mitotic slippage [55]. Cultures treated with TTFields during mitosis possessed significantly increased numbers of large cells many of which possessed multiple nuclei further demonstrating that cells affected by TTFields survived to exit mitosis (See S4 Fig).

These results suggested that cells affected by exposure TTFields during mitosis were compromised in their viability and/or in their ability to proliferate and the possibility of a G_0_ arrest induced by aberrant mitotic exit. Such cell cycle arrest subsequent to aberrant mitotic exit has been shown to be influenced by the cell’s p53 mutational status. In order to test this, we treated asynchronous HCT116 cells that were either p53^+/+^ or p53^-/-^ created by homologous recombination [56] for 24 hours. We then analyzed them for their ability to incorporate BrdU over 2 hours starting 16 hours after removal from the TTFields. The p53^+/+^ TTFields-treated cultures exhibited a significant decrease in BrdU incorporation relative to control cultures while cells deficient in p53 exhibited a smaller decrease in DNA synthesis following exposure to the alternating electric fields (Fig 4D). These results demonstrate that TTFields induced cellular stress that reduced proliferation by inducing a block in G_1_ that was influenced by the p53 status of the cell.

#### Cells exposed to TTFields subsequently exhibit increased apoptosis

**Fig 5:**
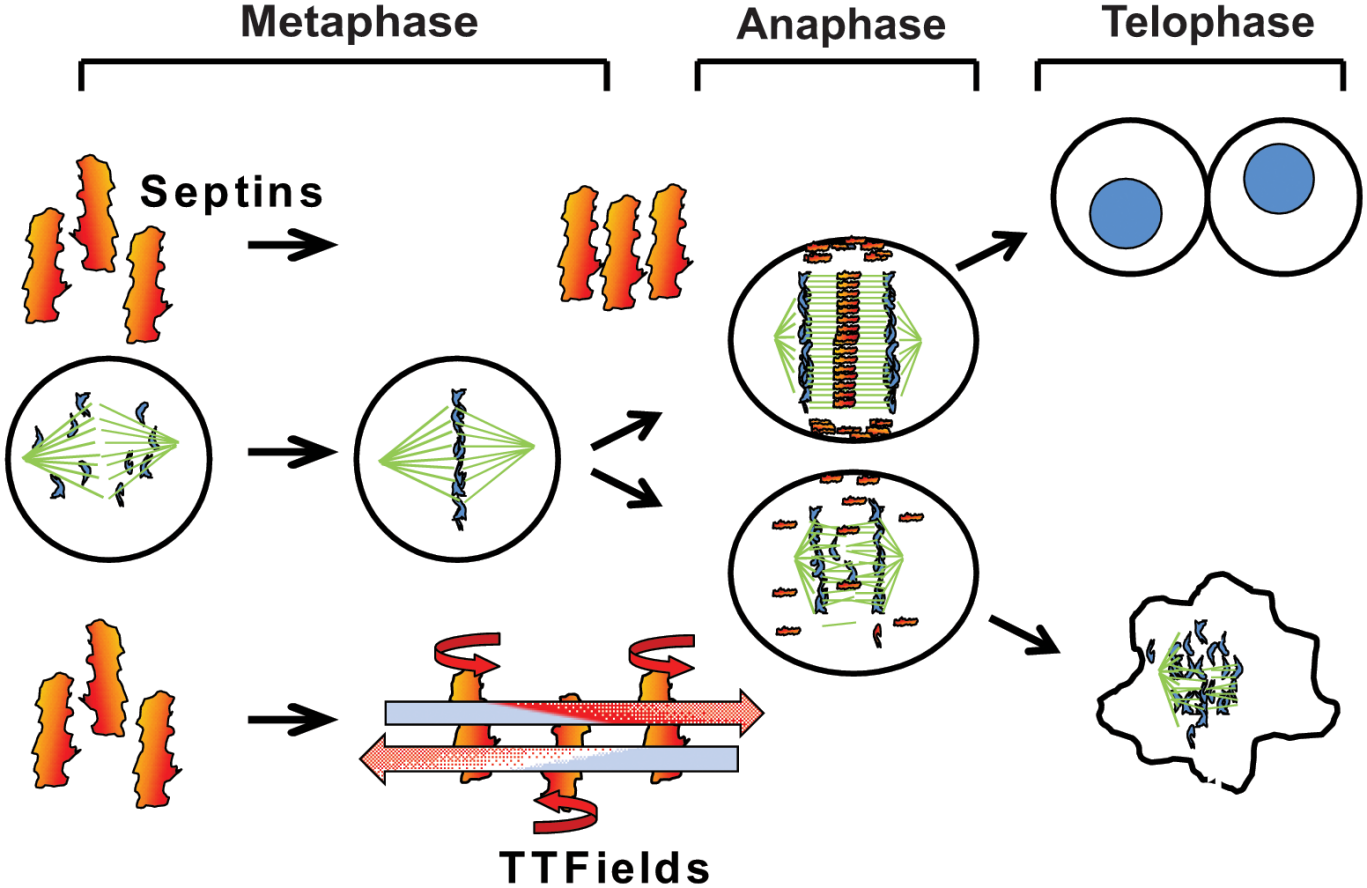
Model for TTFields action leading to mitotic disruption. During mitosis, the Septin 2, 6, 7 complex is recruited to the Anaphase spindle midline and the cytokinetic cleavage furrow by Anillin where it self-assembles into a fibrous lattice due to lateral interactions between parallel Septin filaments. By inducing rotational movement within the parallel fibers at a slightly less more than a right angle to their lateral axis, TTFields are able to inhibit the propagation of lattice formation by disrupting the ability of individual fibers to bind each other. In the absence of proper Septin function, contractile elements of the cytokinetic furrow are not restrained within the equatorial midline of the cell resulting in ectopic furrow malfunction that leads to violent membrane contractions at the onset of anaphase followed by aberrant mitotic exit.

Such G_0/1_ block seen as a result of aberrant mitotic exit is also associated with the induction of apoptosis. We therefore examined whether TTFields treatment of p53^+/+^ HCT116 cells resulted in increased Annexin V staining subsequent to their exposure. Cells were treated for 24 hours and stained for Annexin V while still adherent at 18, 36 and 60 hours after the end of their TTFields exposure. We found that Annexin V binding increased at 18 hours and peaked around 36 hours (Fig 4E). We next asked whether p53^+/+^ cells were more sensitive to TTFields treatment than those lacking p53. We therefore exposed p53^+/+^ and p53^-/-^ HCT116 cells to TTFields for 24 hours and incubated them for an additional 36 hours before staining them with Annexin V. The p53^+/+^ HCT116 cells exhibited a higher percentage of Annexin V positive cells compared to p53^-/-^ HCT116 cells reflecting the differences seen in BrdU incorporation in these cell lines (Fig 4F). Together these data strongly suggest a model whereby TTFields act by perturbing Septin localization and function during mitosis and induce mitotic catastrophe by disrupting the CCF (Fig 5). Cells then experience aberrant mitotic exit, exhibit a G_0_/G_1_ block in cell cycle progression and apoptosis that is influenced by the cells’ p53 mutational status.

## Discussion

Previous studies showed that TTFields perturbed cells in mitosis resulting in plasma membrane contractions and the formation of dynamic blebs on the cell surface. [38, 43]. However, the precise mechanism by which such electric fields-induced forces caused these effects during mitosis remained unknown. We found that while chromosomal migration to the mitotic plate appeared normal, the onset of the membrane blebbing corresponded to the expected time of metaphase exit. The most likely mechanism by which TTFields affect these processes would be by exerting torsional forces on specific proteins that participate in cytokinesis. Previous studies have demonstrated that functional perturbation of proteins that direct the formation and/or regulation of the cytokinetic furrow results in similar plasma membrane blebbing during mitosis [17, 28]. Our claim that TTFields perturb cells coincident with, or just subsequent to metaphase exit is supported by three separate observations: (i) FACS analysis shows that TTFields-treated cells do not show evidence of a block in metaphase exit when compared to paclitaxel treated cells but do experience a delay in mitotic exit, (ii) the timing of the appearance of membrane blebbing is coincident with the expected time of the onset of anaphase and (iii) the absence of observable perturbation in metaphase but presence of cells with disrupted nuclei subsequent to mitosis in TTFields-treated cultures. Thus, when applied therapeutically, this suggests that TTFields act later in the cell cycle than any other anti-cancer interventions which are believed to produce their effects by inducing apoptosis in response to the triggering of G_2_/M or G_1_/S checkpoints.

The regulation and execution of cytokinesis is a complex process that involves the coordination of many proteins acting in concert to rapidly assemble several complex and in highly ordered structures. The CCF is not only highly ordered and spatially regulated, but also must be assembled and activated within a short and precise period near the end of M-phase. Further, the initiation of its ingression must be coordinated with sister chromatid separation during anaphase. Therefore, these combined requirements for both spatial and temporal precision in the execution of these activities makes CCF regulation vulnerable to perturbation by the electromotive forces produced by TTFields.

The specific action of the TTFields within cells relies on their ability to exert rotational and/or torsional stress on a protein or multiple proteins with high dipole moments. Given the evidence that TTFields exposure resulted in ectopic furrow formation we focused on high dipole proteins that also act as regulators of cytokinetic furrow function as potential targets of the TTFields’ anti-tumor activity. The calculation of a protein’s dipole moment requires a complete crystal structure. Unfortunately, no complete crystal structures exist for PLK1, ECT2, Anillin or the centralspindlin heterotetramer. However, using the available crystal structures for the Septin 2, 6 and 7 functional heterotrimer we found that it possessed an unusually large dipole moment of 2711D, which is more than five standard deviations (417.88 D) higher than the average value of 542.66 D derived from the analysis of 14,609 available protein crystal structures [43].

Septins form short filaments that self-assemble into parallel arrays which are non-polar in nature [50, 57, 58]. This is in contrast to actin filaments and microtubules which are long polar polymers to which the charge polarity of their subunits are largely lost. The vector orientation of the GTP-bound Septin heterotrimer’s dipole moment is orthogonal to the long axis of the complex (see Fig 3A). Therefore, the TTFields would be predicted to induce the torsion or rotation of the molecule around this axis. Since the Septin heterotirmeric subunits align parallel to each other within the ordered Septin structures that form during mitosis [57–59], there must be lateral interactions between the fibers that stabilize their lattice like structure. Therefore, TTFields-induced rotation of these fibers likely interferes with the assembly of septins within the midline and CCF. Additionally, these heterotrimers further dimerize into a longer hexamer mediated by end to end interactions between the Septin 2 subunits within each trimer subunit. Significantly, the orientation of the dipole vectors of the two constituent heterotrimers is predicted to be roughly parallel to each other in the same orientation within the resulting hexamer [50]. This arrangement likely results in the creation of a larger aggregate dipole within the resulting hexamer than was calculates for the trimeric subunit.

Septin binds to Anillin and is important for its function during mitosis [20]. ECT2 and Anillin cooperate to stabilize its microtubules within the anaphase spindle [21] and in TTFields treated cells we observe that Septin localization to the anaphase spindle midline was markedly diminished and its microtubules were also disordered. This suggests that the Septin fibrous lattice may play an important role in the stabilization of this structure. The anaphase spindle midline determines both the localization of the CCF and spatially control the activation of non-muscle myosin II that is responsible for driving ingression during anaphase [18]. Therefore, the disordering of the anaphase spindle midline by TTFields is likely to play a major role in producing the observed mitotic catastrophe during anaphase. Indeed, knockdown of Septin 2, 6 or 7 has been shown to result in similar violent membrane blebbing and abnormal mitotic exit [28, 29], supporting that perturbation of Septin function may either partially or fully underlay these phenomena. However, it is not clear what process Septin knockdown perturbs to produce the membrane contractions during mitosis.

The production and regulation of the anaphase spindle and CCF is mechanically complex. It relies on the correct localization of multiple structural and regulatory proteins that serially interact with each other. It is therefore possible that TTFields affect a process, or processes, upstream of Septin recruitment, resulting in the observed decrease in its association with the midline and CCF. However, our staining of PLK1 in TTFields-affected cells clearly shows that the anaphase spindle midline is still present. Therefore, the failure of Septin to localize to the midline is not due to the absence or disruption of this structure but to due to a failure to be recruited and stabilized to it. Also, whereas Anillin is required for Septin localization to mitotic structures [20], MAP-4 is responsible for recruiting the Septin complex to the interphase microtubule cytoskeleton [54]. We showed that Septin 7 re-localization to a newly formed microtubule network is similarly perturbed in a frequency dependent manner by TTFields. Given the different mechanisms of Septin localization to these two structures, our results suggests a direct effect of TTFields on Septins. Together, our observations of frequency dependent alteration of Septin behavior in both mitotic cells and spreading cells provide compelling evidence in support of a model where TTFields perturb cell division by inhibiting the necessary M-phase functions of Septins leading to CCF dysregulation (Fig 5).

It remains possible that other proteins that act within mitosis may also be affected by TTFields and the disruption of mitosis requires combined action on them. For example, the α/β-tubulin dimer, which acts as the functional unit of microtubule polymerization, also possesses a significantly high dipole moment of 1660 D. Since microtubules play a major structural role during mitosis, including chromosomal congression towards the mitotic plate, microtubule capture of chromatid kinetochores the production of the anaphase spindles and the positioning of the CCF, the α/β-tubulin dimer therefore also represents an attractive target of TTFields action [38]. However, in our experiments, beyond the disordering of the anaphase midline microtubules, we were unable to observe gross differences in microtubule structures in fixed mitotic cells prior to anaphase entry or during spreading suggesting that TTFields are unable to directly impact microtubule function. The disordering of the anaphase midline may be either due to a direct effect of TTFields on the microtubules within this structure or due to the observed affect on Septin since similar affects have been reported in ECT2 and Anillin depleted cells [21].

Finally, it has been found that aberrant mitotic exit leads to a p53-dependent G_0/1_ cell cycle arrest leading to apoptosis. This is likely due to a failure to resolve the mitotic spindle apparatus, multiple centrioles and/or the presence of supernumerary chromosomes [37]. Indeed, consistent with this, we have found that cells exposed to TTFields subsequently exhibit decreased proliferation with a failure to enter S phase and increased levels of apoptosis beginning more than 24 hours after TTFields exposure. A p53-dependent G_0/1_ block was observed after cells were exposed to TTFields with apoptosis occurring more than 24 hours after the affected mitosis with levels being greater in p53^+/+^ cells that in the knockouts. This suggests the triggering of a p53-dependant mechanism by TTFields in response to mitotic catastrophe and aberrant mitotic exit. We were unable to detect p53 induction within TTFields-treated cultures (not shown), so the nature of this p53-dependency is unclear. However, these data strongly suggest that the efficacy of patient treatment with TTFields may be also be influenced by tumor genetics.

Together, our observations demonstrate that TTFields affect cell division by interfering with CCF function and that at least one key protein, Septin, involved in this process fails to properly localize to the midline of the anaphase spindle where its binding partners have been shown to be essential for promoting its stability. This results in plasma membrane instability and blebbing that disrupts cytokinesis leading to aberrant mitotic exit and the production of deranged cells that subsequently undergo apoptosis (Fig 5). It is important to note that since TTFields affect cells subsequent to both the G_2_/M and spindle assembly check points; this adds to the unique nature of TTFields when applied in the clinic and may dictate their use with other therapies in the clinic.

## Supporting Information

S1 Fig Serial Extracted Images from Time Lapse Microscopy. Sham-treated (A) and TTFields-treated (B) cells corresponding to Fig 2A and 2B, respectively. 4 minute intervals. Scale bar = 10 μm.
(TIF)

S2 Fig Localization of Non-muscle Myosin II in Sham and TTFields-treated Anaphase Cells. 3D confocal Image reconstruction of anaphase HeLa cells transiently expressing Myosin-IIA-GFP Sham cells (upper teir) and TTFields treated cells (lower teir). DNA was visualized by DAPI. Scale bar = 10 μm.
(TIF)

S3 Fig Serial Extracted Images from Time Lapse Microscopy showing dynamics of membrane contrations. Sham-treated (A) and TTFields-treated (B) cells 4 minute intervals. Scale bar = 10 μm.
(TIF)

S4 Fig TTFields Exposure Leads to Large Cell Formation in MCF-7 Cultures. Images of Sham-treated (**A**) and TTFields-treated (**B**) MCF-7 cells 24 hours after removal treatment showing an increased percentage of large cells (**C**). Scale bar = 50 μm.
(TIF)

S1 Movie Normal Cell Division under Sham Conditions. (AVI)

S2 Movie Abnormal Cell Division with Membrane Contractions under Sham Conditions. (AVI)

S3 Movie Abnormal Cell Division under Conditions of TTFields Exposure with Membrane Contractions. (AVI)

S4 Movie Abnormal Mitotic Exit under Conditions of TTFields Exposure. (AVI)

S5 Movie Mitotic Membrane Blebbing under Sham Conditions. (AVI)

S6 Movie Mitotic Membrane Blebbing under Sham Conditions. (AVI)

S7 Movie Mitotic Membrane Blebbing in cells exposed to TTFields. (AVI)

S8 Movie Mitotic Membrane Blebbing in cells exposed to TTFields. (AVI)
