# Supplementary figures and images for: Tumor Treating Fields Perturb the Localization of Septins and Cause Aberrant Mitotic Exit
Source: PLoS One. 2015 May 26;10(5):e0125269. doi: 10.1371/journal.pone.0125269 (PMC4444126; doi:10.1371/journal.pone.0125269)

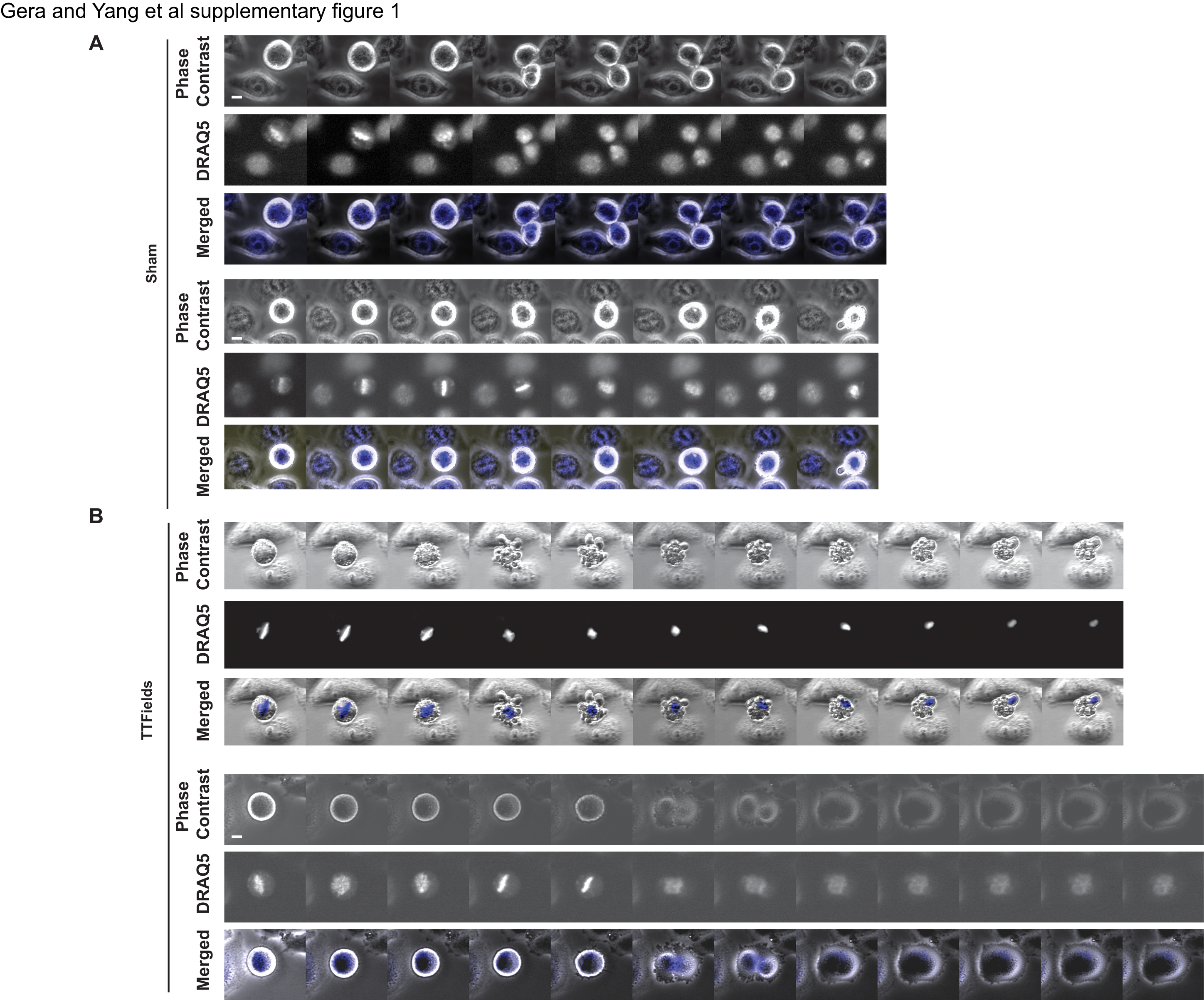

Supplement: S1 Fig — Sham-treated (A) and TTFields-treated (B) cells corresponding to Fig 2A and 2B, respectively. 4 minute intervals. Scale bar = 10 μm. (TIF) [file pone.0125269.s001.tif]

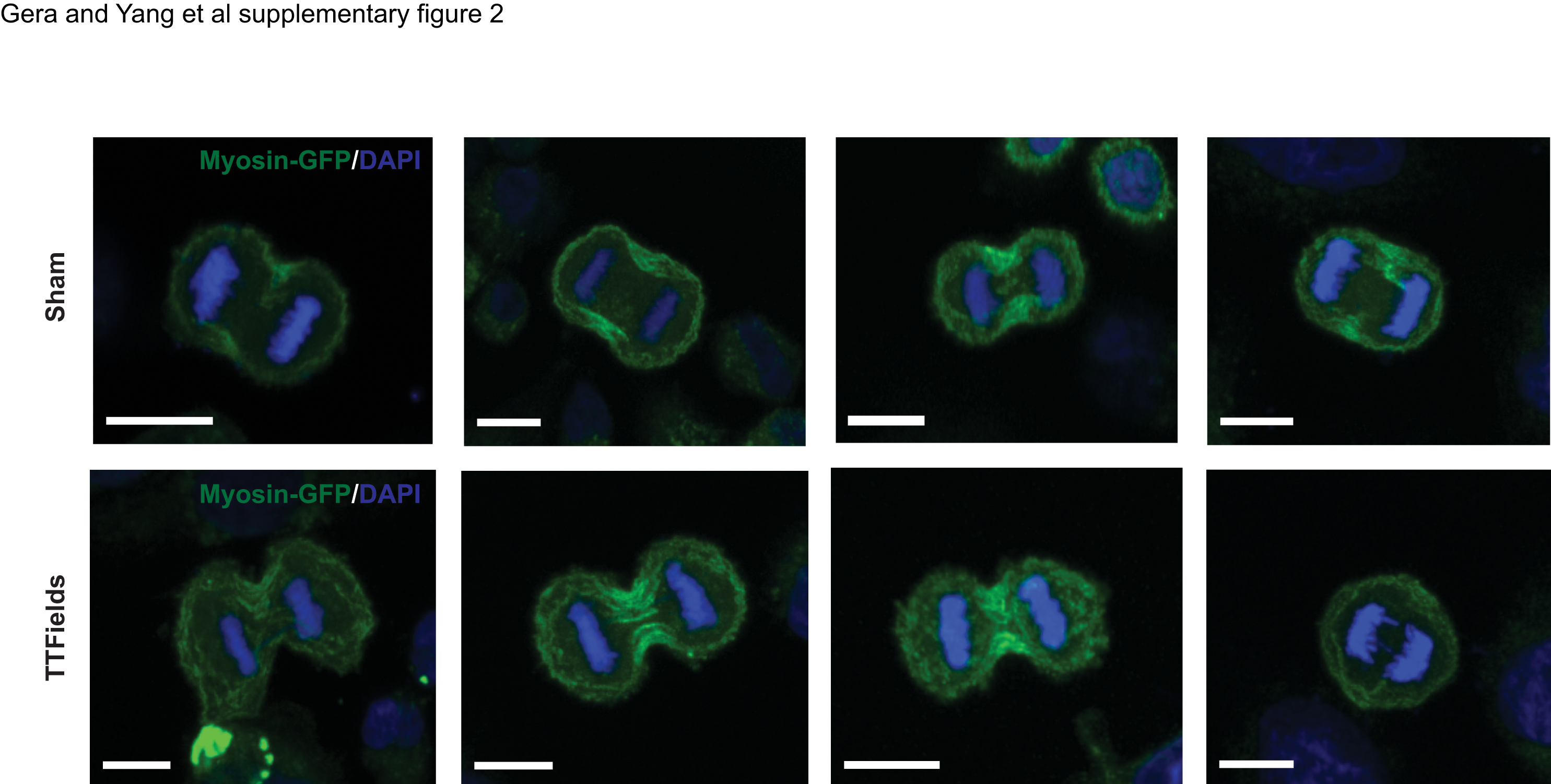

Supplement: S2 Fig — 3D confocal Image reconstruction of anaphase HeLa cells transiently expressing Myosin-IIA-GFP Sham cells (upper teir) and TTFields treated cells (lower teir). DNA was visualized by DAPI. Scale bar = 10 μm. (TIF) [file pone.0125269.s002.tif]

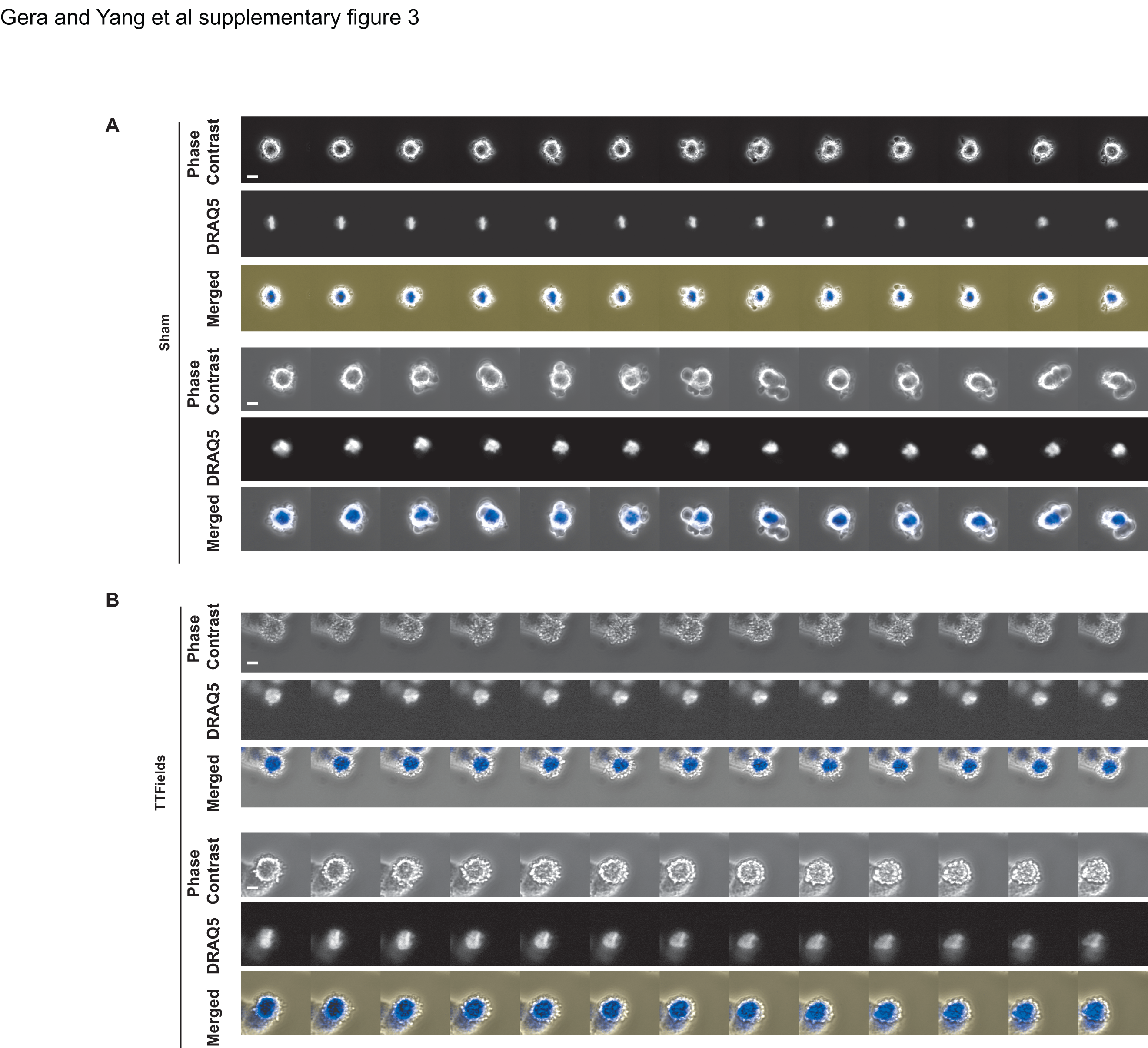

Supplement: S3 Fig — Sham-treated (A) and TTFields-treated (B) cells 4 minute intervals. Scale bar = 10 μm. (TIF) [file pone.0125269.s003.tif]

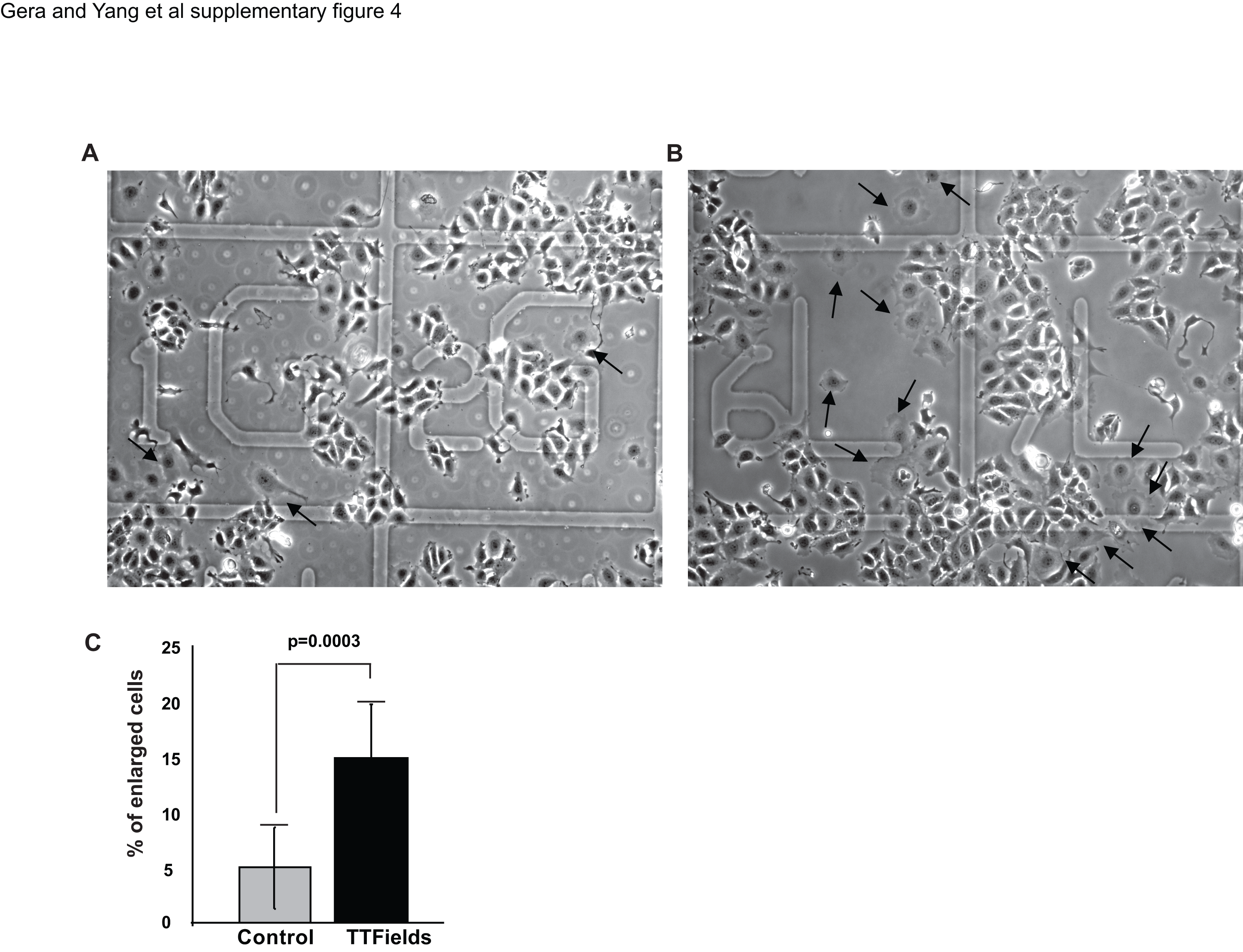

Supplement: S4 Fig — Images of Sham-treated (A) and TTFields-treated (B) MCF-7 cells 24 hours after removal treatment showing an increased percentage of large cells (C). Scale bar = 50 μm. (TIF) [file pone.0125269.s004.tif]
